# Mapping the research of mitochondria and Parkinson’s disease: a bibliometric analysis

**DOI:** 10.3389/fneur.2024.1413762

**Published:** 2024-09-16

**Authors:** Yan-Jun Chen, Ming-Rong Xie, Sheng-Qiang Zhou, Fang Liu

**Affiliations:** ^1^Graduate School of Hunan University of Chinese Medicine, Changsha, China; ^2^National TCM Master Liu Zuyi Inheritance Studio, The Affiliated Hospital of Hunan Academy of Chinese Medicine, Changsha, China; ^3^The First Clinical College of Nanjing University of Chinese Medicine, Nanjing, China

**Keywords:** Parkinson’s disease, mitochondria, bibliometrics, Web of Science, VOSviewer, CiteSpace

## Abstract

**Background:**

Parkinson’s disease (PD) is a chronic, progressive neurodegenerative disorder primarily affecting the elderly. Relevant studies suggest a significant connection between the mitochondria and PD. Publications exploring this connection have steadily increased in recent years. This study employs a bibliometric approach to comprehensively analyze the current status and future directions of the research on mitochondria and PD.

**Method:**

We retrieved data from the Web of Science database and used CiteSpace, VOSviewer, and “Bibliometrix” software to visually analyze various aspects of the research field. These aspects included the number of published papers, contributing countries and institutions, authors, publishing journals, cited references, and keywords.

**Results:**

Our analysis identified a total of 3,291 publications involving 14,670 authors from 2,836 organizations across 78 countries. The publication volume exhibited a continuous upward trend from 1999 to 2023. The United States emerged as the leading force in this research area, contributing the highest number of high-quality publications. Notably, the United States collaborated extensively with Germany and the United Kingdom. The University of Pittsburgh stood out as the most prolific institution. Harvard University had the highest academic influence and closely cooperated with the University of Pittsburgh, Juntendo University, and McGill University. Dr. Hattori Nobutaka was identified as the most prolific author, while Dr. Youle, Richard J emerged as the most influential author based on the highest average citation frequency. The *Journal of Neurochemistry* was the most published journal. The most co-cited paper was titled “*Hereditary early-onset Parkinson’s disease caused by mutations in PINK1.*” The major keywords included oxidative stress, alpha-synuclein, pink1, mitophagy, and mitochondrial dysfunction. Mitofusin 2, ubiquitin, and mitochondrial quality control have been identified as new research hotspots in recent years.

**Conclusion:**

Mitochondria-PD research is experiencing a steady increase in activity, fueled by increasing close collaboration between countries and different institutions. However, there is a need to further strengthen collaboration and communication between developed and developing nations. Current research has focused on the specific mechanisms of mitochondrial dysfunction and their relationship with PD. Mitofusin 2, ubiquitin, and mitochondrial quality control are positioned to be the hotspots and future research directions.

## 1 Introduction

Parkinson’s disease (PD) is a chronic, progressive neurodegenerative disorder characterized by the progressive degeneration of dopaminergic neurons in the brain (1). This degeneration manifests as motor symptoms, such as bradykinesia, rigidity, resting tremor, and postural instability (2). These symptoms progressively worsen, interfering with daily activities and quality of life. PD is a complex condition with multiple contributing factors, including environmental exposures, genetic predisposition, and aging. Despite extensive research, the precise mechanisms underlying the pathogenesis of PD remain unclear, prompting ongoing exploration of new therapeutic targets and strategies.

Mitochondria, as energy-producing organelles, provide essential energy for neuronal activity (3). They are involved in a range of cellular processes, including oxidative phosphorylation (4), calcium signaling (5), and apoptosis (6). In neurons, mitochondria are particularly important as they fuel synaptic transmission and neuronal survival. Mitochondrial dysfunction has been implicated in numerous neurological diseases, with PD being a prime example (7). The relationship between mitochondria and PD has attracted significant attention in recent years. Mitochondrial dysfunction can result in dopaminergic neuronal degeneration (8), a key factor in PD pathogenesis. Additionally, PD patients exhibit abnormalities in mitochondrial morphology, function, and dynamics (9), further strengthening the link between mitochondrial dysfunction and PD. Targeting mitochondria for novel treatments holds promise as a potential effective therapeutic approach for PD. With the development of aging societies, research on the link between mitochondria and PD has gradually become a major focus. While numerous studies have been published, a comprehensive analysis of the current research status and development frontiers in the field of mitochondria and PD is lacking.

Bibliometric analysis, a systematic statistical analysis method (10), offers a powerful tool for effectively exploring knowledge structures and identifying research hotspots and future directions. This is made possible through the visualization of data and network collaboration maps (11). In this study, we utilized bibliometric analysis to systematically examine the relationship between mitochondria and PD. We aimed to identify influential authors, key research themes, and emerging trends within this rapidly evolving field. By doing so, we aimed to contribute to a more profound understanding of the role of mitochondria in the pathogenesis of PD.

## 2 Methods

### 2.1 Search strategy and data sources

**Figure 1:**
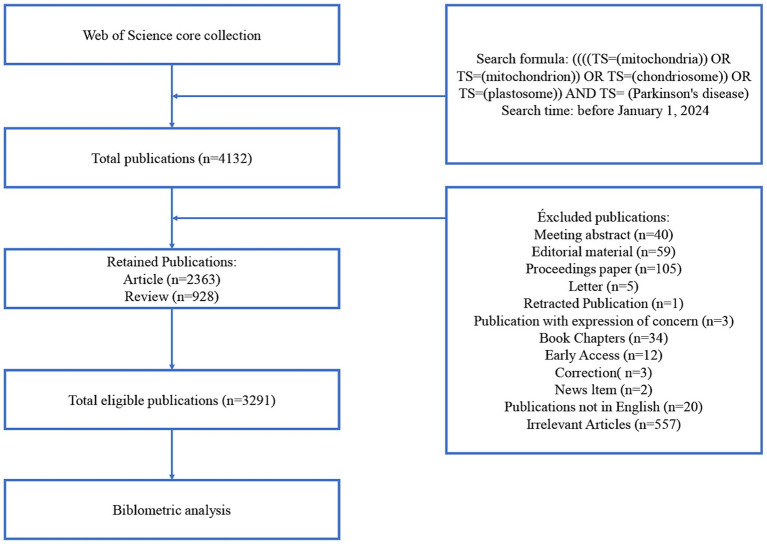
Flowchart of literature selection.

We used the Web of Science (WoS) database, a comprehensive and authoritative literature retrieving tool encompassing literature from various scientific disciplines, for data retrieval. WoS is considered to be the most suitable database for bibliometric analysis (12). Data were collected from the WoS core collection. Specific filters were applied using the WoS topic search function (Topics, TS). The search formula was ((((TS = (mitochondria)) OR TS = (mitochondrion)) OR TS = (chondriosome)) OR TS = (plastosome)) AND TS = (Parkinson’s disease). To ensure that the most up-to-date data was included, the search timeframe was set before January 1, 2024. Additionally, the search was restricted to articles and reviews published in English. Meeting abstracts, proceedings papers, editorial material, and other types of publications were excluded. It was speculated that although the initial topic search may contain ostensibly related literature, it can also include marginally related or irrelevant literature. To address this, two members of the research team checked and analyzed the literature, manually screening to exclude irrelevant literature, resulting in a final dataset of 3,291 publications for analysis. The literature screening process is illustrated in Figure 1.

### 2.2 Data analysis

CiteSpace, VOSviewer, and “Bibliometrix” software were used for the bibliometric analysis. These tools hold much significance in analyzing literature visualization. They are also recognized and used by most scholars. CiteSpace is a practical literature software for quantitative analysis, which can present the structure and distribution of scientific knowledge (13). VOSviewer can visually analyze the knowledge in the research field and provides multiple types of view interpretation (14). Bibliometrix is an R package that collates relevant literature in the field and visualizes the results (15).

## 3 Results

### 3.1 Scientific publications related to PD and mitochondria

A total of 3,291 publications were involved in this study, including 2,363 articles (71.8%) and 928 reviews (28.2%). These publications were written by 14,670 authors from 2,836 organizations in 78 countries. They were published in 625 journals and cited 121,199 references from 7,996 journals.

### 3.2 Temporal trend of publication outputs

**Figure 2:**
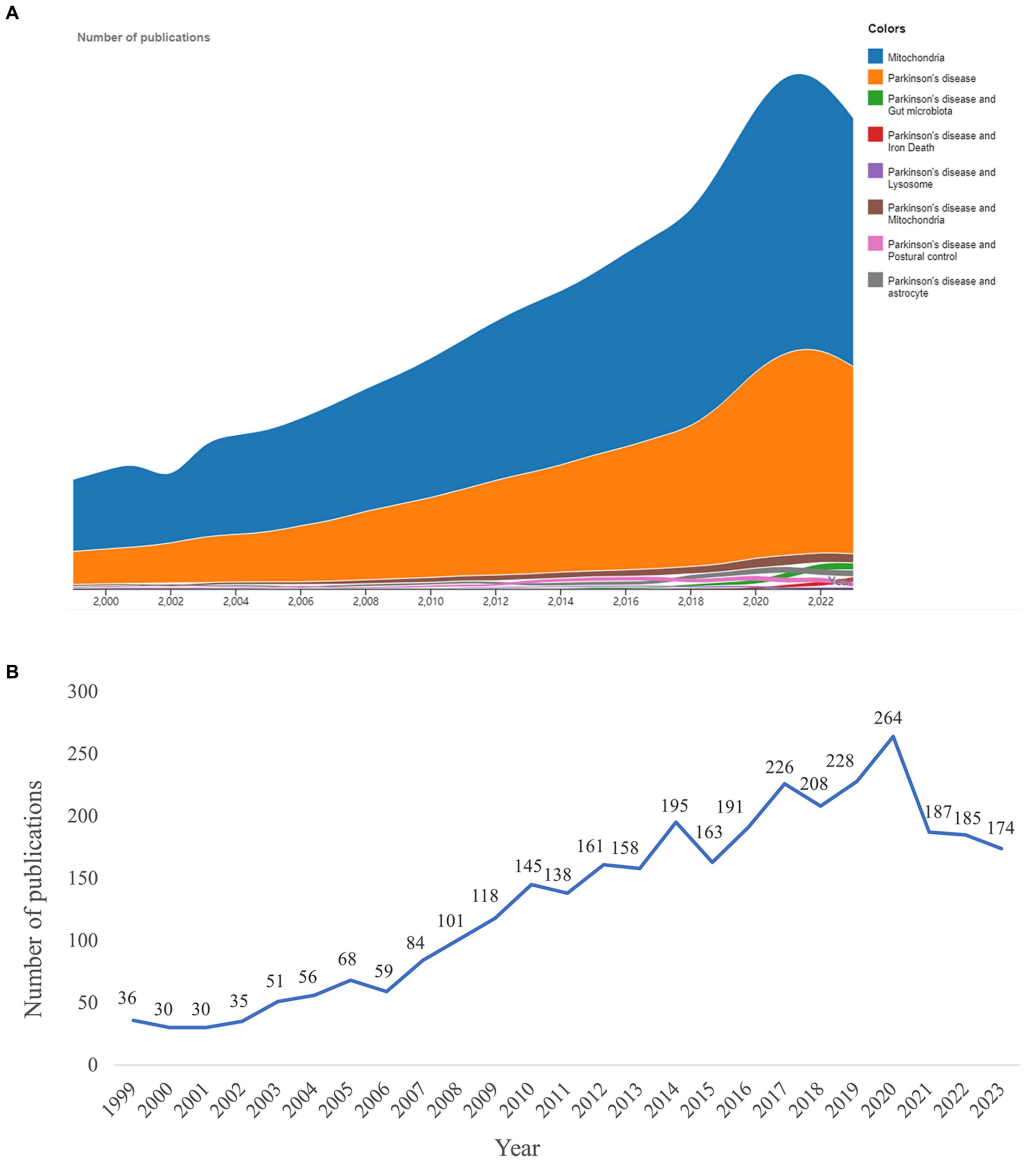
Research results. **(A)** Number of publications on mitochondria and Parkinson’s disease (PD) was compared with other topics. **(B)** Trend in the number of publications on mitochondria and PD from 1999 to 2023.

The number of publications reflected the research trends. In the investigated time frame, publications on mitochondria and PD increased significantly compared with publications on other PD-related research topics (Figure 2A). From 1999 to 2023, a total of 3,921 publications were published on mitochondria and PD, with the number of publications showing an upward trend (Figure 2B). The time trend change can be divided into two periods. In the first period, from 1999 to 2007, the number of publications increased slowly, with fewer than 100 publications each year. In the second period, from 2008 to 2023, the number of publications increased rapidly, with a peak of 264 publications in 2020.

### 3.3 Geographic distribution and national collaboration

**Figure 3:**
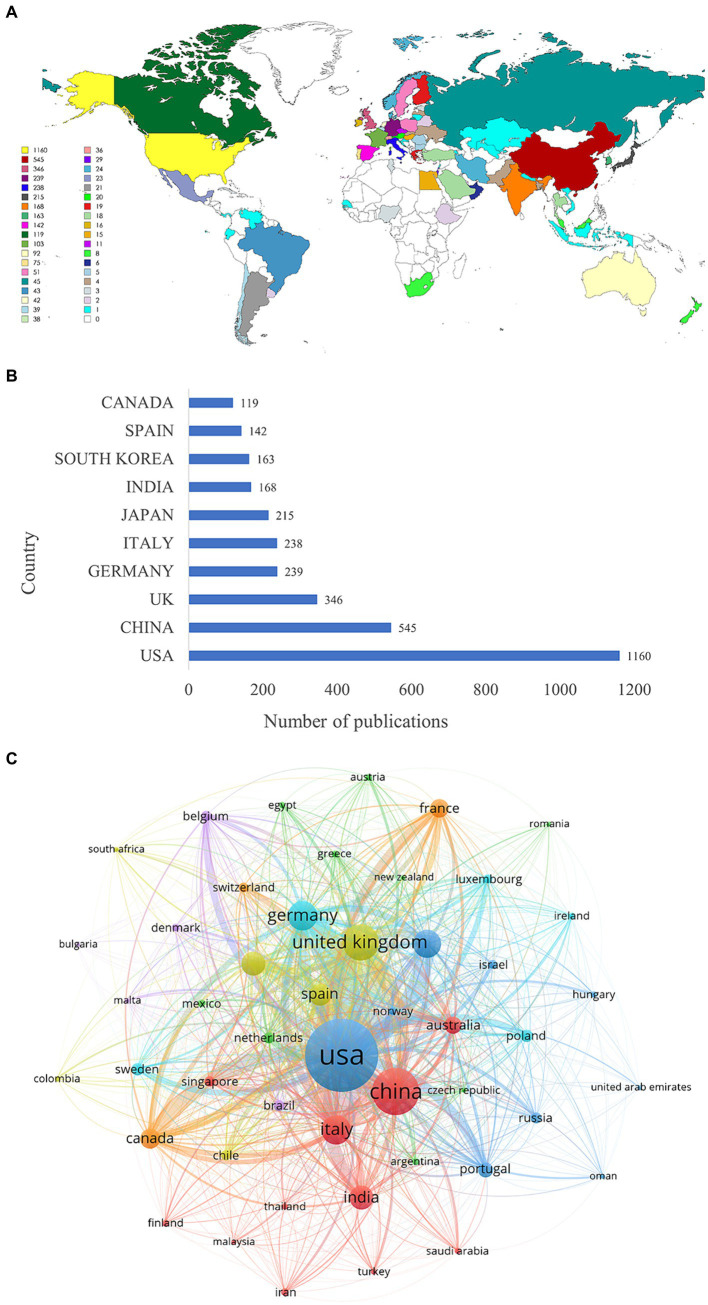
Analysis of countries. **(A)** Geographic distribution of countries. **(B)** Top 10 countries in terms of the number of publications. **(C)** The map of collaboration network between countries. The thicker the line, the closer the collaboration.

The heat map of the geographical distribution of publications revealed a concentration of research activity in North America, Asia, and Europe, with notable collaboration among these regions (Figure 3A). Researchers from 78 countries have contributed to the field of mitochondria and PD research. Among these countries, the United States ranked first (1,160 publications), followed by China (545 publications) and the United Kingdom (346 publications) (Figure 3B). The United States emerged as the leading force in this research area, contributing the highest number of high-quality publications and demonstrating strong collaborative ties with Germany and the United Kingdom (Figure 3C).

### 3.4 Research organizations

**Figure 4:**
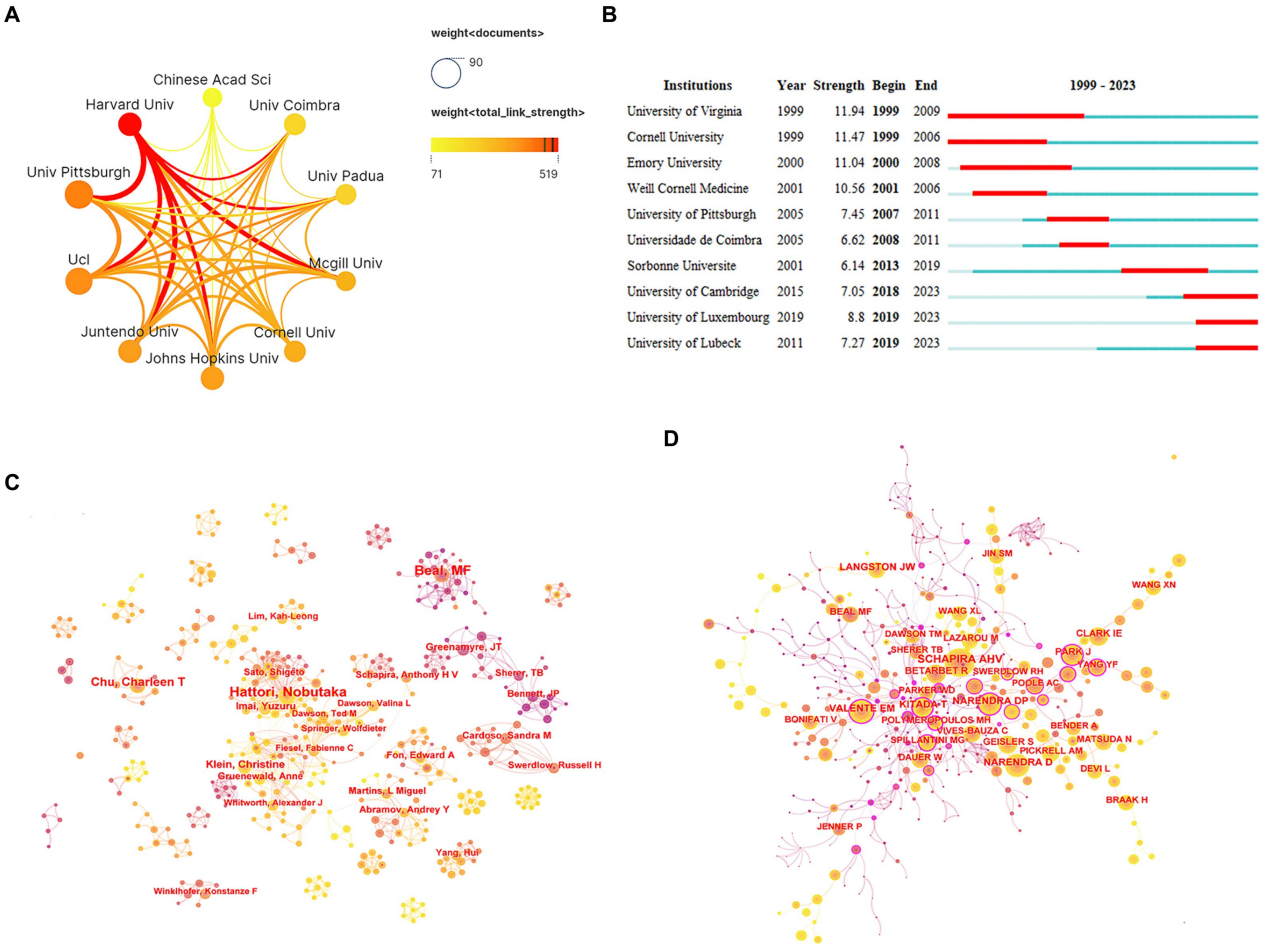
Analysis of organizations and authors. **(A)** Network collaboration map of top 10 organizations. **(B)** Top 10 organizations with the strongest citation bursts. **(C)** Author collaboration network diagram. **(D)** Co-cited author collaboration network diagram.

In terms of the number of publications, four of the top 10 organizations were located in the United States. The University of Pittsburgh topped the list with 76 publications, followed by University College London (69 publications), Harvard University (52 publications), and Johns Hopkins University (52 publications) (Table 1). Harvard University had the highest academic influence and maintained close collaboration with the University of Pittsburgh, Juntendo University, and McGill University (Table 1; Figure 4A). In organizational research outbreaks, the University of Cambridge, the University of Luxembourg, and the University of Lubeck have been identified as emerging and active organizations in recent years (Figure 4B).

**Table 1:** The top 10 productive institutions.

Rank	Institution	Documents	Citations	Average number of citations
1	University of Pittsburgh	76	7,626	100.34
2	University College London	69	7,898	114.46
3	Harvard University	52	9,951	191.37
4	Johns Hopkins University	52	8,701	167.33
5	Juntendo University	51	5,335	104.61
6	University of Coimbra	45	2,310	51.33
7	Cornell University	44	7,589	172.48
8	University of Padua	41	1,489	36.32
9	McGill University	37	5,337	144.24
10	Chinese Academy of Sciences	36	1,602	44.5

### 3.5 Authors and co-cited authors

An analysis of the authors associated with publications can identify prominent scholars and key forces within the research field. Dr. Hattori Nobutaka emerged as the most prolific author with 42 papers, followed by Dr. Beal, M. Flint (40 papers) and Dr. Chu, Charleen T (28 papers). These highly productive authors all benefited from stable collaboration networks and research teams (Figure 4C). Dr. Youle, Richard J stood out as the most influential author based on the highest average citation frequency (879.67 citations/literature) (Table 2). Notably, two teams—one comprised of Dr. Cardoso, Sandra M and Dr. Swerdlow, Russell H, and the other comprised of Dr. Greenamyre, JT, and Dr. Sherer, TB—demonstrated close collaboration and have published high-quality articles.

**Table 2:** Top 15 authors.

Rank	Author	Documents	Citations	Average number of citations	Country	Institution
1	Dr. Hattori, Nobutaka	42	4,179	99.5	Japan	Juntendo University
2	Dr. Beal, Myron Flint	40	6,833	170.83	USA	Weill Cornell Medicine Feil Family Brain and Mind Research Institute
3	Dr. Chu, Charleen T	28	2,512	89.71	USA	University of Pittsburgh School of Medicine
4	Dr. Imai, Yuzuru	18	1932	107.33	Japan	Juntendo University Graduate School of Medicine
5	Dr. Klein, Christine	18	839	46.61	Germany	University of Lubeck
6	Dr. Cardoso, Sandra Morais	17	835	49.12	Portugal	Universidade de Coimbra
7	Dr. Lu, Bingwei	16	2,302	143.88	USA	Stanford University
8	Dr. Fon, Edward A	16	3,237	202.31	Canada	McGill University
9	Dr. Martins, L. Miguel	16	918	57.38	UK	University of Cambridge
10	Dr. Yang, Hui	16	830	51.88	China	Capital Medical University
11	Dr. Youle, Richard J	15	13,195	879.67	USA	NIH National Institute of Neurological Disorders & Stroke
12	Dr. Sato, Shigeto	15	3,120	208	Japan	Juntendo University
13	Dr. Schapira, Anthony H., V	15	2,966	197.73	UK	University College London
14	Dr. Greenamyre, J. Timothy	15	6,446	429.73	USA	University of Pittsburgh
15	Dr. Gruenewald, Anne	15	827	55.13	Luxembourg	University of Luxembourg

Co-cited authors refer to instances where two or more authors are cited together in the same publications. Among the top 10 most-cited authors, seven authors were cited over 500 times. Dr. Schapira, Ahv was the most cited author (1,401 times), followed by Dr. Narendra, Derek P (1,109 times) and Dr. Beal, M. Flint (610 times) (Table 3; Figure 4D).

**Table 3:** Top 10 co-cited authors.

Rank	Author	Citations	Country	Institution
1	Dr. Schapira, Anthony H. V	1,401	UK	Institute of Neurology, University College London
2	Dr. Narendra, Derek P	1,109	USA	NIH National Institute of Neurological Disorders & Stroke
3	Dr. Beal, Myron Flint	610	USA	Weill Cornell Medicine Feil Family Brain and Mind Research Institute
4	Dr. Valente, Enza Maria	609	Italy	University of Pavia
5	Dr. Kitada, Tohru	584	Japan	Ottawa Hospital Research Institute
6	Dr. Langston, John William	535	USA	Parkinson’s Institute
7	Dr. Betarbet, R	492	USA	Emory University
8	Dr. Parks, Janice K	489	USA	University of Virginia
9	Dr. Parker, W. Drew	454	USA	University of Georgia
10	Dr. Swerdlow, Russell Howard	443	USA	University of Kansas

### 3.6 Journals and co-cited journals

**Figure 5:**
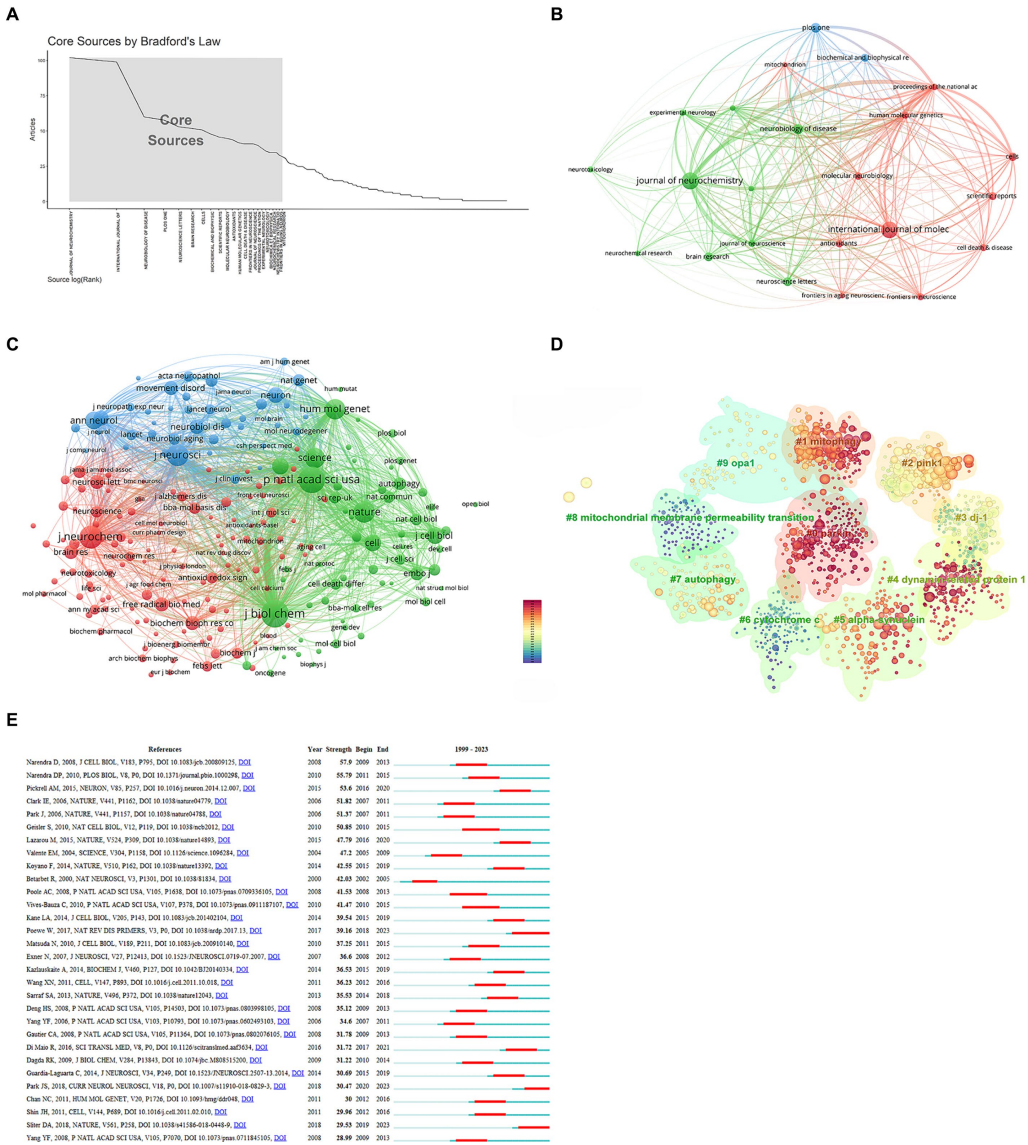
Analysis of journals and co-cited references. **(A)** Core journals. **(B)** Interconnections of core journals. **(C)** Network map of co-cited journals. **(D)** Cluster analysis of co-cited literature. **(E)** Top 30 references with the strongest citation bursts.

A Bradford’s law analysis (16) identified 23 core journals in the field of mitochondria and PD research (Figure 5A). These core journals were primarily concentrated in neurology and biology fields (Figure 5B), with 1,107 publications, accounting for 33.64% of total publications. The top three core journals with the most publications were the *Journal of Neurochemistry* (102 publications, 3.1%), *International Journal of Molecular Sciences* (99 publications, 3.01%), and *Neurobiology of Disease* (60 publications, 1.8%) (Table 4). It is noteworthy that the *Journal of Neurochemistry* has the highest quality of literature with an average citation frequency of 106.22.

**Table 4:** List of the core journals.

Rank	Source	Documents	Citations	Average number of citations	IF	JCR
1	Journal of Neurochemistry	102	10,834	106.22	4.7	Q2
2	International Journal of Molecular Sciences	99	1,639	16.56	5.6	Q1
3	Neurobiology of Disease	60	4,305	71.75	6.1	Q1
4	Plos One	58	3,816	65.79	3.7	Q2
5	Neuroscience Letters	53	2,612	49.28	2.5	Q3
6	Brain Research	52	2,541	48.87	3.8	Q2
7	Cells	51	1,144	22.43	6	Q2
8	Biochemical and Biophysical Research Communications	48	1894	39.46	3.1	Q3
9	Scientific Reports	46	1778	38.65	4.6	Q2
10	Molecular Neurobiology	45	2,525	56.11	5.1	Q2

IF, impact factor; JCR, journal citation reports.

A co-cited journal network analysis is presented in Figure 5C. The effect of a journal is closely related to the number of citations. The top three co-cited journals were the *Journal of Biological Chemistry* (12,086 citations), *Proceedings of the National Academy of Sciences of the United States of America* (10,868 citations), and the *Journal of Neurochemistry* (8,054 citations) (Table 5).

**Table 5:** Top 10 cited journals.

Rank	Source	Citations	IF	JCR
1	Journal of Biological Chemistry	12,086	4.8	Q2
2	Proceedings of the National Academy of Sciences of the United States of America	10,868	11.1	Q1
3	Journal of Neurochemistry	8,054	4.7	Q2
4	Journal of Neuroscience	7,200	5.3	Q1
5	Nature	6,814	64.8	Q1
6	Human Molecular Genetics	6,137	3.5	Q3
7	Science	5,866	56.9	Q1
8	Annals of Neurology	4,541	11.2	Q1
9	Journal of Cell Biology	3,961	7.8	Q1
10	Cell	3,875	64.5	Q1

IF, impact factor; JCR, journal citation reports.

### 3.7 Co-cited references and reference bursts

Co-cited references formed the basis of the research field. The top 10 cited references are listed in Table 6. The most co-cited paper (503 citations) was “*Hereditary early-onset Parkinson’s disease caused by mutations in PINK1*,” published in the journal *Science* with an impact factor of 56.9. This was followed by “*Parkin is recruited selectively to impaired mitochondria and promotes their autophagy*” (455 citations) and “*Mutations in the parkin gene cause autosomal recessive juvenile parkinsonism*” (433 citations). A cluster analysis of co-cited literature was performed using CiteSpace. The color of nodes in the cluster from dark blue to yellow and red reveals the shift in research focus over time (Figure 5D). While “mitochondrial membrane permeability transition” and “cytochrome” received attention in the past, recent research has shifted toward “parkin,” “dynamin-related protein 1,” and “mitophagy”.

**Table 6:** Top 10 co-cited references.

Rank	Title	Type	Citation times	Year	Journal	IF	JCR
1	Hereditary early-onset Parkinson’s disease caused by mutations in PINK1	Article	503	2004	Science	56.9	Q1
2	Parkin is recruited selectively to impaired mitochondria and promotes their autophagy	Article	455	2008	Journal of Cell Biology	7.8	Q1
3	Mutations in the parkin gene cause autosomal recessive juvenile parkinsonism	Article	433	1998	Nature	64.8	Q1
4	PINK1 Is Selectively Stabilized on Impaired Mitochondria to Activate Parkin	Article	413	2010	Plos Biology	9.8	Q1
5	Chronic systemic pesticide exposure reproduces features of Parkinson’s disease	Article	377	2000	Nature Neuroscience	25	Q1
6	Drosophila pink1 is required for mitochondrial function and interacts genetically with parkin	Article	376	2006	Nature	64.8	Q1
7	Mitochondrial dysfunction in Drosophila PINK1 mutants is complemented by parkin	Article	350	2006	Nature	64.8	Q1
8	PINK1/Parkin-mediated mitophagy is dependent on VDAC1 and p62/SQSTM1	Article	329	2010	Nature Cell Biology	21.3	Q1
9	Chronic Parkinsonism in humans due to a product of meperidine-analog synthesis	Article	325	1983	Science	56.9	Q1
10	Mitochondrial complex I deficiency in Parkinson’s disease.	Article	312	1989	Lancet	169	Q1
10	Mutation in the alpha-synuclein gene identified in families with Parkinson’s disease	Article	312	1997	Science	56.9	Q1

IF, impact factor; JCR, journal citation reports.

Reference bursts reflect the number of citations within a specific timeframe. Among the strongest reference bursts, the most highly cited articles in recent years were “*Parkinson’s disease*,” “*Parkin and PINK1 mitigate STING-induced inflammation*,” and “*Mitochondrial dysfunction in Parkinson’s disease: New mechanistic insights and therapeutic perspectives*” (Figure 5E).

### 3.8 Keywords

**Figure 6:**
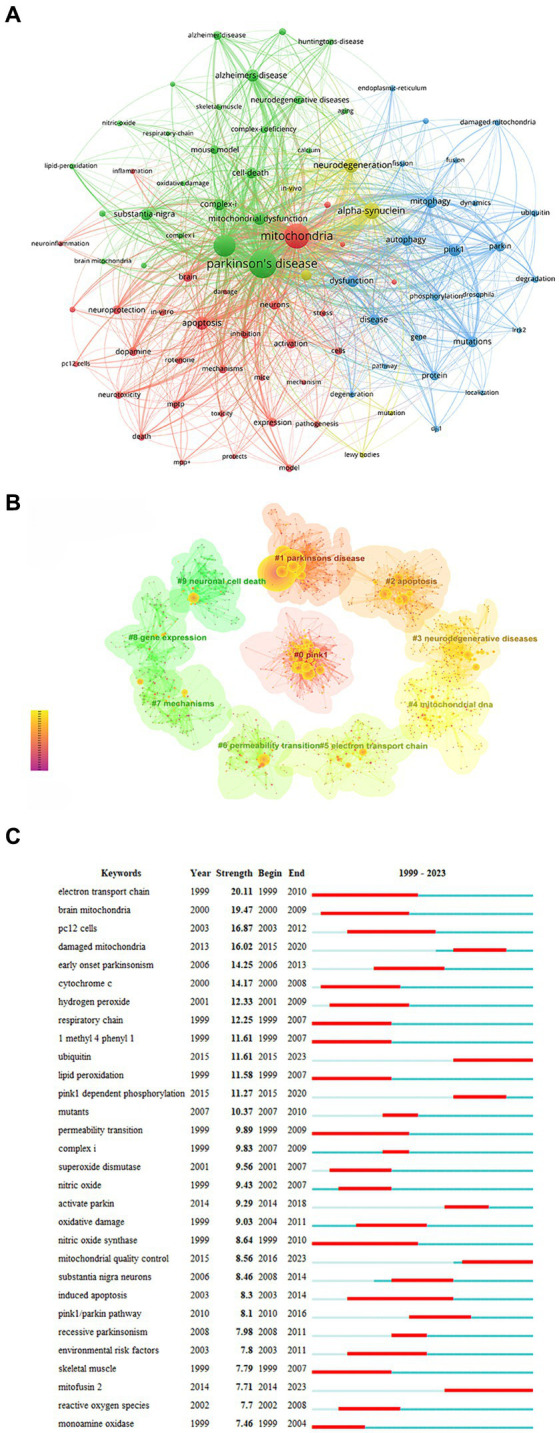
Analysis of keywords. **(A)** Network map of keywords. **(B)** Cluster analysis of keywords. **(C)** Top 30 keywords with the strongest citation.

Keyword analysis helps identify research hotspots and key areas within a research field. In the field of mitochondria and PD, frequently occurring keywords included oxidative stress (1,188 times), alpha-synuclein (677 times), neurodegeneration (520 times), apoptosis (436 times), pink1 (403 times), mitophagy (390 times), autophagy (352 times), and mitochondrial dysfunction (361 times) (Figure 6A). Keyword cluster analysis revealed that “pink1,” “apoptosis,” and “Parkinson’s disease” clusters were prominent research topics attracting significant scholarly attention in recent years (Figure 6B).

Keywords with the strongest bursts identify phrases that appear frequently within a short period, reflecting shifts in research focus over time. The top three bursts were “electron transport chain” (strength = 20.11), “brain mitochondria” (strength = 19.47), and “damaged mitochondria” (strength = 16.87) (Figure 6C). “Electron transport chain,” “nitric oxide synthase,” and “induced apoptosis” have been the longest-lasting hot spots in the last two decades. The keywords that broke out in the past 3 years primarily focus on “mitofusin 2,” “ubiquitin,” and “mitochondrial quality control.”

## 4 Discussion

In recent years, the relationship between mitochondria and PD has gradually attracted considerable attention from the scientific community. Furthermore, publications on mitochondria and PD have grown significantly compared with publications in other PD-related research areas within the investigated time frame. Therefore, it is necessary to comprehensively analyze the progress and hotspot changes in this research field to provide an intuitive understanding to researchers. To analyze publications related to mitochondria and PD, we used bibliometric analysis, aiming to further explore the development of this research field and predict emerging trends.

The number of publications can reflect the development of the current research field. From 1999 to 2023, publications on mitochondria and PD steadily increased. This upward trend suggests a potential link between the development of this research field and the acceleration of the aging population. As societies age, exploration of the mechanisms of geriatric diseases such as PD has attracted increasing attention. A geographical analysis revealed a concentration of research activity in North America, Asia, and Europe, with notable collaborations among these regions. The top three countries in terms of the number of publications were the United States, China, and the United Kingdom. The United States was the main force in research with the highest quality of publications. Four of the top 10 organizations were from the United States. Harvard University had the highest academic influence and closely collaborated with the University of Pittsburgh, Juntendo University, and McGill University. The main research forces were concentrated in developed countries, so it is particularly important to strengthen academic communication and collaboration between developed and developing countries to promote the development of this research field.

In terms of authors, Dr. Hattori Nobutaka was the most prolific author (42 papers), followed by Dr. Beal, M. Flint (40 papers), and Dr. Chu, Charleen T (28 papers). Dr. Hattori Nobutaka comes from Juntendo University. His primary research area is Neurosciences & Neurology. In 2010, Dr. Hattori Nobutaka and his research team discovered that PINK1 recruited Parkin to damaged mitochondria for mitochondrial degradation in a membrane potential-dependent manner (17). Afterward, in 2020, they found that loss of Parkin led to mitochondrial accumulation and that the impairment of mitochondrial clearance may be the cause of dopaminergic neuron loss (18). In the same year, they discovered that mitochondrial *UQCRC1* mutations contributed to familial parkinsonism (19). In recent years, their main research has focused on the gene *PRKN* (20) and proteins related to mitochondrial function, such as FBXO7 (21) and CHCHD2 (22). It is noteworthy that Dr. Youle, Richard J is the most influential author with the highest average citation frequency of literature (879.67 citations/literature). Dr. Youle, Richard J comes from NIH’s National Institute of Neurological Disorders and Stroke. His main research areas are cell biology, neuroscience, and neurology. In 2011, Dr. Youle, Richard J and his research team elaborated on the mechanism of mitophagy and its link with PD, which attracted great attention in the academic community (23). In 2012, they explored mitochondrial fission, fusion, and stress, while also further describing the association of mitochondrial fission, fusion, and stress with PD (24). Subsequently, in 2018, they found that PINK1 and Parkin mitigated inflammation in PD by mediating mitophagy (25). With regards to group collaboration, the team of Dr. Cardoso, Sandra M and Dr. Swerdlow, Russell H explored the effect of mitochondrial dysfunction on α-synuclein (26, 27). Dr. Greenamyre, JT and Dr. Sherer, TB focused on rotenone models of PD concerning mitochondrial dysfunction (28–30). Their findings not only provided important insights into the role of mitochondria in the pathogenesis of PD but also provided new ideas for treatment and drug development. These research efforts have significantly influenced the research direction of mitochondria and PD, which has promoted the progress and development of the entire research field.

Concerning journals, the *Journal of Neurochemistry* was the most widely published (102 articles, 3.1%), with the highest average number of citations (106.22), followed by the *International Journal of Molecular Sciences* (99 articles, 3.01%) and *Biology of Neurological Diseases* (60 articles, 1.8%). The top three co-cited journals were the *Journal of Biological Chemistry* (12,086 citations), followed by *Proceedings of the National Academy of Sciences of the United States of America* (10,868 citations) and the *Journal of Neurochemistry* (8,054 citations). Among the top 10 cited journals, seven were located in JCR Q1 with high quality. Cited journals comprised the basis of citing journals, which provided references to support the current research.

Co-cited references formed the basis of the research field. The literature “*Hereditary early-onset Parkinson’s disease caused by mutations in PINK1*” was the most co-cited paper (503 citations), followed by “*Parkin is recruited selectively to impaired mitochondria and promotes their autophagy*” (455 citations) and “*Mutations in the parkin gene cause autosomal recessive juvenile parkinsonism*” (433 citations). These top-cited articles focusing on mutations in *PINK1* and *Parkin* genes and their interactions represent the foundational research on mitochondria in PD.

Keywords represent the core research content of the literature. Keyword analysis can quickly reflect the hotspot distribution and focus in the research field. Beyond PD and mitochondria, frequently occurring keywords included oxidative stress, alpha-synuclein, neurodegeneration, PINK1, mitophagy, and mitochondrial dysfunction. Oxidative stress is a key player in PD pathogenesis. It elevates reactive oxygen species (ROS) within cells, which can directly damage mitochondria and contribute to the degeneration of dopaminergic neurons (31). Alpha-synuclein, the major component of Lewy bodies, a hallmark of PD pathology, accumulates abnormally (32). This aggregation can exacerbate mitochondrial oxidative stress (33) and increase ROS production (31), which leads to neurodegeneration. Mutations in the *PINK1* gene are a frequent cause of autosomal recessive PD (34). Localized to the mitochondria, PINK1 regulates the balance between mitochondrial fission and fusion (35). Additionally, PINK1 acts upstream of Parkin, recruiting Parkin to the mitochondria to initiate autophagy in damaged mitochondria (36). Senescent and damaged mitochondria are eliminated by mitophagy, and the PINK1/Parkin pathway plays a crucial role in this process (37). Mitochondrial dysfunction manifests primarily as abnormalities in mitochondrial morphological structure and function, including mitochondrial number abnormalities, mitochondrial DNA damage, and mitochondrial-related protein abnormalities. Research has revealed a close association between mitochondrial DNA variations, complex I deficiency, and PD (38). Moreover, a related study has revealed that genetic abnormalities In *Parkin*, *PINK1*, *DJ-1*, and *LRRK2* could lead to mitochondrial dysfunction and were associated with monogenic PD (39). These keywords reflected that the focus of research was concentrated on the specific mechanisms of mitochondrial dysfunction and their relationship with PD. The study of genes, proteins, and pathways related to mitochondrial function remains the mainstream direction of the present research.

The core themes and domain structure can be quickly identified from the massive literature through the keyword cluster analysis of the literature, and the research and development of this field can be further understood. In recent years, “pink1,” “apoptosis,” and “Parkinson's disease” clusters have been important research topics in this field with regard to cluster analysis. Dr. Klein, Christine, Dr. Valente, Enza Maria, and Dr. Muqit, Miratul M K contributed to the “pink1” research topic. Dr. Maruyama, Wakako and Dr. Naoi, Makoto made many contributions to the topic of “apoptosis.”

Animal models of PD mainly included the mouse model, rat model, *Caenorhabditis elegans* model, drosophila model, zebrafish model, and monkey model. Rotenone, 6-OHDA, and MPTP were the commonly used drugs in the process of construction of animal models of PD (40–42). The SH-SY5Y neuroblastoma cells and primary neuronal cultures are widely used for the cell model (43). Mitochondria-targeted biomimetic nanoparticles can promote neuronal mitochondrial biogenesis by regulating the NAD/SIRT1/PGC-1α/PPARγ/NRF1/TFAM pathway, thereby ameliorating mitochondrial dysfunction and alleviating PD symptoms (44). Clinical studies are also exploring gene therapy, drug therapy, and other methods to improve mitochondrial function to further treat PD. Gene therapy has been used to treat mitochondrial dysfunction in PD, mainly including enhancing dopamine synthesis (45), improving neurotrophic factors expression (46), and in the regulation of the human basal ganglia circuits (47). Coenzyme Q10, with its antioxidant properties, has demonstrated potential in reducing oxidative stress by protecting the mitochondrial membrane (48). Studies have suggested that PD patients with enrichment of mitochondrial gene risk variants may benefit from mitochondrial enhancer of coenzyme Q10 treatment (49). Oral administration of nicotinamide riboside (NR) can increase the level of nicotinamide adenine dinucleotide (NAD), thereby mitigating mitochondrial dysfunction and alleviating PD symptoms (50). Clinical trials have demonstrated great tolerability for NR treatment, with evidence of elevated levels of NAD in the brains of patients (51). The development of drugs that specifically target mitochondria holds promise as a potential treatment strategy for PD and warrants further investigations in larger clinical trials.

In recent years, popular research topics have mainly included mitofusin 2, ubiquitin, and mitochondrial quality control. These research hotspots may serve both as the latest research trends and as future research directions. Mitofusin 2 is a mitochondrial outer membrane protein, which is involved in the mitochondrial dynamic process (52). Loss of mitofusin 2 can lead to damage of the mitochondrial respiratory chain (53). It has also been demonstrated that mitofusin 2 had an imperative role in axonal mitochondrial transport (54) and axonal projections of dopaminergic neurons (55). Ubiquitination targets mitochondrial proteins for degradation through mitophagy (56). Parkin, for instance, can ubiquitinate certain candidate substrate proteins, tagging them for proteasomal degradation (57). Mitochondrial fission, fusion, mitophagy, and mitochondrial biogenesis are involved in mitochondrial quality control, and they coordinate with each other to regulate the quantity and quality of mitochondria (58). Defective mitochondrial quality control has been implicated in the development of PD (59). The identification of these emerging research hotspots has helped to reveal the research dynamics of mitochondria and PD, paving the way for future advancements in our understanding and treatment of the disease.

This study has some limitations. First, the analysis was limited to review articles and original research publications in English. This approach may have excluded relevant research published in other languages or formats. Second, with the continuous update of the WoS database, the included publications may differ slightly from the actual number of publications.

## 5 Conclusion

The bibliometric analysis of mitochondria and PD provides valuable insight into the current research status and development trend. Research related to mitochondria and PD is expanding considerably. There is growing collaboration among different countries, institutions, and authors. However, the collaboration and exchange between developed and developing countries needs to be further strengthened. Current research has focused on the specific mechanisms of mitochondrial dysfunction and their relationship with PD. Emerging research hotspots will likely include mitofusin 2, ubiquitin, and mitochondrial quality control, which are likely to shape future research directions in this field.

## Data availability statement

The original contributions presented in the study are included in the article/supplementary material, further inquiries can be directed to the corresponding authors.

## Author contributions

Y-JC: Software, Visualization, Writing – original draft. M-RX: Software, Writing – original draft. S-QZ: Writing – review & editing. FL: Software, Visualization, Writing – review & editing.
